# Delamanid, linezolid, levofloxacin, and pyrazinamide for the treatment of patients with fluoroquinolone-sensitive multidrug-resistant tuberculosis (Treatment Shortening of MDR-TB Using Existing and New Drugs, MDR-END): study protocol for a phase II/III, multicenter, randomized, open-label clinical trial

**DOI:** 10.1186/s13063-018-3053-1

**Published:** 2019-01-16

**Authors:** Myungsun Lee, Jeongha Mok, Deog Kyeom Kim, Tae Sun Shim, Won-Jung Koh, Doosoo Jeon, Taehoon Lee, Seung Heon Lee, Ju Sang Kim, Jae Seuk Park, Ji Yeon Lee, Song Yee Kim, Jae Ho Lee, Kyung-Wook Jo, Byung Woo Jhun, Young Ae Kang, Joong Hyun Ahn, Chang-Ki Kim, Soyoun Shin, Taeksun Song, Sung Jae Shin, Young Ran Kim, Heejung Ahn, Seokyung Hahn, Ho Jeong Won, Ji Yeon Jang, Sang Nae Cho, Jae-Joon Yim

**Affiliations:** 1 Clinical Research Section, International Tuberculosis Research Center, 247, Jangchungdan-ro, Jung-gu, Seoul, 04564 Republic of Korea; 2 Division of Pulmonology, Allergy and Critical Care Medicine, Department of Internal Medicine, Pusan National University Hospital, 179, Gudeok-ro, Seo-gu, Busan, 49241 Republic of Korea; 3 Division of Pulmonary and Critical Care Medicine, Department of Internal Medicine, Seoul National University Boramae Medical Center, 20, Boramae-ro 5-gil, Dongjak-gu, Seoul, 07061 Republic of Korea; 4 Division of Pulmonary and Critical Care Medicine, Department of Internal Medicine, Asan Medical Center, 88, Olympic-ro 43-gil, Songpa-gu, Seoul, 05505 Republic of Korea; 5 Division of Pulmonary and Critical Care Medicine, Department of Medicine, Samsung Medical Center, Sungkyunkwan University School of Medicine, 81, Irwon-ro, Gangnam-gu, Seoul, 06351 Republic of Korea; 6 Division of Pulmonary and Critical Care Medicine, Department of Internal Medicine, Pusan National University Yangsan Hospital, 20, Geumo-ro, Mulgeum-eup, Yangsan-si, Gyeongsangnam-do 50612 Republic of Korea; 7 Department of Pulmonology, Ulsan University Hospital, University of Ulsan College of Medicine, 877, Bangeojinsunhwando-ro, Dong-gu, Ulsan, 44033 Republic of Korea; 8 Department of Pulmonology, Korea University Ansan Hospital, 123, Jeokgeum-ro, Danwon-gu, Ansan, Gyeonggi-do 15355 Republic of Korea; 9 Department of Pulmonary and Critical Care Medicine, Department of Internal Medicine, The Catholic University of Korea, Incheon St. Mary’s Hospital, 56, Dongsu-ro, Bupyeong-gu, Incheon, 21431 Republic of Korea; 10 Division of Pulmonary and Critical Care Medicine, Department of Internal Medicine, Dankook University Hospital, 201, Manghyang-ro, Dongnam-gu, Cheonan-si, Chungcheongnam-do 31116 Republic of Korea; 11 Division of Pulmonary and Critical Care Medicine, Department of Internal Medicine, National Medical Center, 245, Eulji-ro, Jung-gu, Seoul, 04564 Republic of Korea; 12 Division of Pulmonology, Department of Internal Medicine, Institute of Chest Diseases, Severance Hospital, Yonsei University College of Medicine, 50-1, Yonsei-ro, Seodaemun-gu, Seoul, 03722 Republic of Korea; 13 Division of Pulmonary and Critical Care Medicine, Department of Internal Medicine, Seoul National University Bundang Hospital, 82, Gumi-ro 173beon-gil, Bundang-gu, Seongnam-si, Gyeonggi-do 13620 Republic of Korea; 14 Seoul Clinical Laboratories, 13, Heungdeok 1-ro, Giheung-gu, Yongin, Gyeonggi-do 16954 Republic of Korea; 15 Laboratory Medicine Center, The Korean Institute of Tuberculosis, 168-5, Osongsaengmyeong 4-ro, Osong-eup, Heungdeok-gu, Cheongju-si, Chungcheongbuk-do 28158 Republic of Korea; 16 Institute of Infectious Disease and Molecular Medicine, University of Cape Town, Rondebosch 7701, Cape Town, South Africa; 17 Department of Microbiology, Brain Korea 21 PLUS Project for Medical Science, Yonsei University College of Medicine, 50-1, Yonsei-ro, Seodaemun-gu, Seoul, 03722 Republic of Korea; 18 Medical Research Collaborating Center, Seoul National University Hospital, 101, Daehak-ro, Jongno-gu, Seoul, 03080 Republic of Korea; 19 Division of Pulmonary and Critical Care Medicine, Department of Internal Medicine, Seoul National University College of Medicine, 103, Daehak-ro, Jongno-gu, Seoul, 03080 Republic of Korea

**Keywords:** Tuberculosis, Multidrug-resistant tuberculosis, Multicenter randomized trial, Non-inferiority, Shorter regimen, Delamanid, Linezolid

## Abstract

**Background:**

Treatment success rates of multidrug-resistant tuberculosis (MDR-TB) remain unsatisfactory, and long-term use of second-line anti-TB drugs is accompanied by the frequent occurrence of adverse events, low treatment compliance, and high costs. The development of new efficient regimens with shorter treatment durations for MDR-TB will solve these issues and improve treatment outcomes.

**Methods:**

This study is a phase II/III, multicenter, randomized, open-label clinical trial of non-inferiority design comparing a new regimen to the World Health Organization-endorsed conventional regimen for fluoroquinolone-sensitive MDR-TB. The control arm uses a conventional treatment regimen with second-line drugs including injectables for 20–24 months. The investigational arm uses a new shorter regimen including delamanid, linezolid, levofloxacin, and pyrazinamide for 9 or 12 months depending on time to sputum culture conversion. The primary outcome is the treatment success rate at 24 months after treatment initiation. Secondary outcomes include time to sputum culture conversion on liquid and solid media, proportions of sputum culture conversion on liquid media after 2 and 6 months of treatment, treatment success rate according to pyrazinamide resistance, and occurrence of adverse events grade 3 and above as evaluated by the Common Terminology Criteria for Adverse Events. Based on an α = 0.025 level of significance (one-sided test), a power of 80%, and a < 10% difference in treatment success rate between the control and investigational arms (80% vs. 70%) when the anticipated actual success rate in the treatment group is assumed to be 90%, the number of participants needed per arm to show non-inferiority of the investigational regimen was calculated as 48. Additionally, assuming the proportion of fluoroquinolone-susceptible MDR-TB among participants as 50%, and 5% loss to follow-up, the number of participants is calculated as *N*/( 0.50 × 0.95), resulting in 102 persons per group (204 in total).

**Discussion:**

This trial will reveal the effectiveness and safety of a new shorter regimen comprising four oral drugs, including delamanid, linezolid, levofloxacin, and pyrazinamide, for the treatment of fluoroquinolone-sensitive MDR-TB. Results from this trial will provide evidence for adopting a shorter and more convenient treatment regimen for MDR-TB.

**Trial registration:**

ClincalTrials.gov, NCT02619994. Registered on 2 December 2015.

**Electronic supplementary material:**

The online version of this article (10.1186/s13063-018-3053-1) contains supplementary material, which is available to authorized users.

## Background

Multidrug-resistant tuberculosis (MDR-TB) is a disease caused by *Myocbacterium tuberculosis* that is resistant to at least isoniazid and rifampicin, the two most important anti-TB drugs. It accounts for 3.5% of newly diagnosed TB patients and 20.5% of retreatment TB patients globally. In 2016, approximately 490,000 people were diagnosed with MDR-TB, approximately 9% of whom had extensively drug-resistant tuberculosis (XDR-TB), which is resistant to isoniazid and rifampicin plus any fluoroquinolone and at least one of three injectable second-line drugs [1, 2]. In South Korea, 852 new patients with MDR-TB were notified in 2016, and this number has been relatively unchanged in recent years [3, 4].

Traditionally, in order to treat MDR-TB, second-line anti-TB drugs must be used for at least 20 months. Additionally, an injectable drug is needed for the first 8 months of treatment [5]. Despite the long duration of treatment, the treatment success rate remains unsatisfactory. According to the latest World Health Organization (WHO) report, the proportion of MDR-TB/rifampicin-resistant TB (RR-TB) patients who successfully completed treatment (i.e., were cured or completed treatment) was as low as 54% [2]. One important reason for disappointing treatment outcomes is non-adherence to treatment; 15% of MDR-TB patients were lost to follow-up before treatment completion [2].

Patients with MDR-TB frequently experience adverse events (AEs) over the treatment course. One report showed that 57.3% of 5346 patients experienced at least one adverse drug event (ADE) including gastrointestinal disorders (32.1%), ototoxicity (14.6%), and psychiatric disorders (13.2%) [6]. Additionally, among 1519 patients who developed ADEs with available data on MDR-TB therapy, 70.4% required a change of MDR-TB treatment [6]. Another report from a Korean TB cohort showed that major ADEs were more frequent in patients being treated with second-line regimens (16%) compared with first-line regimens (2.5%) [7].

Considering all of these issues, a shorter and more convenient regimen for MDR-TB treatment is urgently needed. Fortunately, repurposed anti-TB drugs including linezolid or newly developed drugs including delamanid and bedaquiline are available for patients with MDR-TB [8–11]. Recently, TB research has focused on identifying the optimal combination of existing and new anti-TB drugs to improve treatment outcomes as well as shorten treatment duration [12].

We hypothesize that a new regimen consisting of fully oral medications including delamanid, linezolid, levofloxacin, and pyrazinamide for 9–12 months is non-inferior to the conventional regimen including second-line anti-TB drugs for 20–24 months to treat MDR-TB in terms of treatment outcomes.

## Methods/design

### Setting

**Fig. 1:**
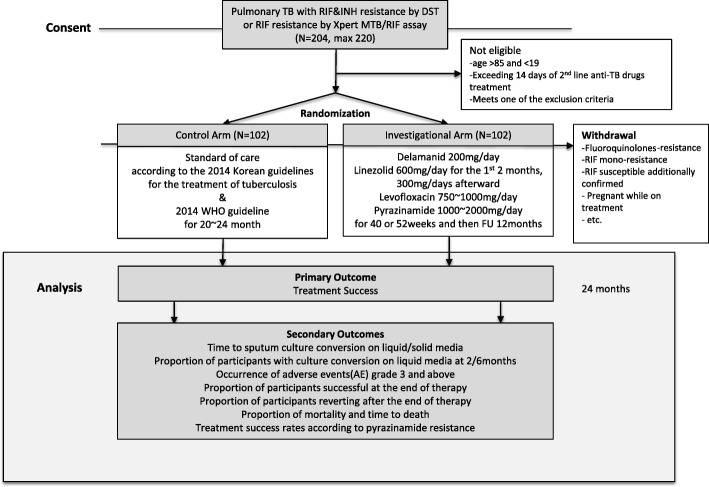
The flow diagram of the trial

This randomized controlled trial is being conducted at 12 referral hospitals in South Korea. The flow diagram for the trial is shown in Fig. 1. Patients with pulmonary TB satisfying the inclusion criteria are competitively enrolled by the investigators in the 12 participating hospitals.

### Design

This is a phase II/III, multicenter, randomized, open-label clinical trial with two arms. Adult pulmonary TB patients with confirmed MDR-TB or RR-TB are eligible. Once eligibility is confirmed and the participant consents to participate, he/she will be randomly assigned to one of the two groups (arm 1 or 2) at a 1:1 ratio.

#### Arm 1 (control arm)

Treatment will be performed according to the 2014 Korean guidelines for the treatment of tuberculosis [13, 14] and the 2014 WHO guidelines [15] (Table 1). Treatment principles include the following: (1) intensive phase regimen comprising four effective second-line anti-TB drugs (including an injectable) and pyrazinamide should be used for a minimum of 8 months duration; (2) at least five active drugs should be used, appropriately selected based on drug susceptibility testing results (e.g., pyrazinamide, an injectable, a fluoroquinolone, prothionamide, and cycloserine); (3) efforts should always be made to use a fluoroquinolone (preferentially levofloxacin or moxifloxacin); (4) prothionamide or cycloserine should be used preferentially, but when it is not possible, *p*-aminosalicylic acid (PAS) can be used; (5) the recommended length of the intensive phase including injectable is 8 months and the total treatment duration is 20–24 months.

**Table 1:** Dose and schedule of anti-TB drugs recommended by the Korean national guidelines [37]

Drug	Dose		Usage
	Usual dose	Maximal dose	
Pyrazinamide	1000 mg (< 50 kg) 1500 mg (50–70 kg) 2000 mg (> 70 kg)	2000 mg	Once, before or after meal
Kanamycin Amikacin Streptomycin Capreomycin	15 mg/kg (< 50 years) 10 mg/kg (≥ 50 years)	1000 mg (< 50 years) 750 mg (≥ 50 years)	Once, intramuscularly
Cycloserine	500 mg (< 50 kg) 750 mg (50–70 kg) 750–1000 mg (> 70 kg)	1000 mg	Once or divide in two, before or after meal
Prothionamide	500 mg (< 50 kg) 750 mg (50–70 kg) 750–1000 mg (> 70 kg)	1000 mg	Once or divide in two, after meal
*p*-aminosalicylic acid	150 mg/kg	12 g	Divide in two, after meal
Levofloxacin	750 mg (< 50 kg) 1000 mg (50–70 kg)	1000 mg	Once, before or after meal
Moxifloxacin	400 mg	400 mg	Once, before or after meal

#### Arm 2 (investigational arm)

The regimen consists of fully oral medications including delamanid, linezolid, levofloxacin, and pyrazinamide for 9–12 months (Table 2). Treatment principles include the following: (1) delamanid will be used for the entire treatment period unless it must be stopped for reasons such as AEs; (2) linezolid is started at a dose of 600 mg/day for 2 months and reduced to 300 mg/day in the third month of treatment or earlier when AEs occur; (3) levofloxacin can be substituted with moxifloxacin in case of AE development, and this substitution will not be considered as a drug change since both are fluoroquinolones of similar potency; (4) pyrazinamide can be continued even if pyrazinamide resistance is discovered on the phenotypic drugs susceptibility testing after commencing treatment [15]; (5) total treatment duration is 9 months (40 weeks) when sputum culture conversion occurs within 3 months of treatment or 12 months (52 weeks) when sputum culture conversion occurs between 3 and 6 months of treatment.

**Table 2:** Dose and schedule of anti-TB drugs of investigational arm

Drug	Dose	Usage
Linezolid	600 mg/day for initial 2 months, then 300 mg/day	Once
Delamanid	200 mg/day	Divide in two, together with meal
Levofloxacin	750 mg (< 50 kg) 1000 mg (50–70 kg)	Once, before or after meal
Pyrazinamide	1000 mg (< 50 kg) 1500 mg (50–70 kg) 2000 mg (> 70 kg)	Once

The study timeline is shown in Tables 3 and 4. Treatment adherence is evaluated during every visit by a research nurse who keeps track of packages and returned drugs. If scheduled visits are delayed or cancelled, the study team will try to contact participants as soon as possible by telephone.

**Table 3:** Study timeline for control arm

	Screening	Baseline visit^a^	Treatment						End of treatment^g^ (End of study)
Weeks (w)	–2 w ~ –1 day	0	1 w	2 w	4 w	8 w	12 w ~ (every 4w)	52 w ~ (every 2 months)	80 w ~ 24 months
Visit window	NA	NA	± 4 days	± 4 days	± 1w	± 2w	± 2 w	± 2 w	± 2 w
Consent	X								
Randomization		X							
Medical history	X								
Physical exam	X		X	X	X	X	X	X	X
Neurological exam	X								
Sputum AFB smear	X ^e^	X ^e^	X	X	X	X	X	X	X
TB culture (solid)	X ^e^	X ^e^	X	X	X	X	X	X	X
TB culture (liquid)	X ^e^	X ^e^	X	X	X	X	X	X	X
Genotypic DST	If available								
Phenotypic DST^b^	With first/reverted cultured *Mycobacterium tuberculosis*								
CXR	X ^e^	X ^e^	X ^h^	X ^h^	X	X	X	X	X
Chemistry, electrolytes	X ^e^	X ^e^	X	X	X	X	X	X	X
Complete blood count	X ^e^	X ^e^	X	X	X	X	X	X	X
ECG	X ^e^								
Urine HCG^c^	X	X							
HIV, HBV^d^		X ^e^							
Optic test	X								
Compliance of drug intake			X	X	X	X	X	X	X
Adverse drug reaction			X	X	X	X	X	X	X
Other medication^f^	X	X	X	X	X	X	X	X	X

*AFB* acid-fast bacilli, *CXR* chest *x*-ray, *DST* drug susceptibility testing, *ECG* electrocardiogram, *HBV* hepatitis B virus, *HCG* human chorionic gonadotropin, *HIV* human immunodeficiency virus, *NA* not applicable, *TB* tuberculosis

^a^Administration of anti-TB regimen can begin at baseline visit since drug-resistant TB must be treated immediately

^b^Drug susceptibility test for isoniazid, rifampicin, ethambutol, pyrazinamide, streptomycin, kanamycin, amikacin, capreomycin, ofloxacin, levofloxacin, moxifloxacin, prothionamide, cycloserine, and *p*-aminosalicylic acid (can be omitted for patients with results already provided)

^c^Only in females of childbearing potential (blood HCG test results are available)

^d^Study drugs can be administered before obtaining the results since eligibility is not determined by these results

^e^Can be omitted if previous tests except ECG were done within 4 weeks. In case of ECG, within 1 week

^f^Check prohibited drugs for exclusion criteria at screening visit and check immunosuppressants including steroids after enrollment

^g^End of treatment visit will be determined by the time of culture conversion

^h^Can be omitted if previous test was done within 2 weeks

**Table 4:** Study timeline for investigational arm

	Screening	Baseline visit^a^	Treatment					End of treatment (EOT)^g^	EOT to end of study
Weeks (w)	–2 w ~ –1 d	0	1 w	2 w	4 w	8 w	12 w ~ (every 4w)	40 w ~ 52 w	~ 24 months (every 2 months)
Visit window	NA	NA	± 4 days	± 4 days	±1w	±2w	±2 w	±2 w	±2 w
Consent	X								
Randomization		X							
Medical history	X								
Physical exam	X		X	X	X	X	X	X ^g^	
Neurological exam	X		X	X	X	X	X	X ^g^	
Sputum AFB smear	X ^e^	X ^e^	X	X	X	X	X	X	X
TB culture (solid)	X ^e^	X ^e^	X	X	X	X	X	X	X
TB culture (liquid)	X ^e^	X ^e^	X	X	X	X	X	X	X
Genotypic DST	If available								
Phenotypic DST^b^	With first/reverted cultured *Mycobacterium tuberculosis*								
Resistance test	If needed (for linezolid, delamanid)								
CXR	X ^e^	X ^e^	X ^h^	X ^h^	X	X	X	X	X
Chemistry, electrolytes	X ^e^	X ^e^	X	X	X	X	X	X ^g^	
Complete blood count	X ^e^	X ^e^	X	X	X	X	X	X ^g^	
ECG	X ^e^	X ^e^	X	X	X	X	X	X ^g^	
Urine HCG^c^	X	X	X	X	X	X	X	X ^g^	
HIV, HBV^d^		X ^e^							
Optic test	X	X	X	X	X	X	X	X ^g^	
Compliance of drug intake			X	X	X	X	X	X ^g^	
Adverse drug reaction			X	X	X	X	X	X ^g^	
Other medication^f^	X	X	X	X	X	X	X	X ^g^	

*AFB* acid-fast bacilli, *CXR* chest *x*-ray, *DST* drug susceptibility testing, *ECG* electrocardiogram, *HBV* hepatitis B virus, *HCG* human chorionic gonadotropin, *HIV* human immunodeficiency virus, *NA* not applicable, *TB* tuberculosis

^a^Administration of anti-TB regimen can begin at baseline visit since drug-resistant TB must be treated immediately

^b^Drug susceptibility test for isoniazid, rifampicin, ethambutol, pyrazinamide, streptomycin, kanamycin, amikacin, capreomycin, ofloxacin, levofloxacin, moxifloxacin, prothionamide, cycloserine, and *p*-aminosalicylic acid (can be omitted for patients with results already provided)

^c^Only in females of childbearing potential (blood HCG test results are available)

^d^Study drugs can be administered before obtaining the results since eligibility is not determined by these results

^e^Can be omitted if previous tests except ECG were done within 4 weeks. In case of ECG, within 1 week

^f^Check prohibited drugs for exclusion criteria at screening visit and check immunosuppressants including steroids after enrollment

^g^End of study visit will be determined by the time of culture conversion. Omit test after end of treatment visit

^h^Can be omitted if previous test was done within 2 weeks

### Outcomes

The primary outcome is treatment success rate at 24 months after the initiation of treatment. The secondary outcomes include time to sputum culture conversion to negative on liquid and solid culture media, proportion of participants with sputum culture conversion at 2 and 6 months of treatment on liquid culture medium, occurrence of AEs grade 3 and above, proportion of participants with treatment success at the end of treatment, proportion of participants reverting to positive sputum culture after the end of treatment, treatment success rates according to pyrazinamide resistance, proportion of deaths, and time to death.

### Definitions

#### Culture conversion and reversion

We define sputum culture conversion as two consecutive negative sputum cultures taken at least 4 weeks apart. The date of culture conversion is defined as the date of the initial negative culture. Culture conversion was also defined as a patient who could not expectorate sputum after one negative sputum culture. When two or more positive cultures occur again after negative conversion, it is regarded as reversion.

#### Treatment outcomes

We define treatment outcomes with reference to 2014 WHO Guidelines [15] (Table 5). For the investigational group, 6 months will be considered as the intensive phase. Furthermore, “stopping” of drugs during treatment will not be considered as “drug change.”

**Table 5:** Definitions of treatment outcomes for patients who are drug-resistant

Cured	Treatment completed as recommended by the national policy without evidence of failure AND culture conversion after the intensive phase^a^
Treatment completed	Treatment completed as recommended by the national policy without evidence of failure BUT no record of culture conversion after the intensive phase^a^
Treatment failed	Treatment terminated or need for permanent regimen change of at least two anti-TB drugs because of: •Lack of conversion by the end of the intensive phase^a^; or •Bacteriological reversion in the continuation phase after conversion to negative; or •Evidence of additional acquired resistance to fluoroquinolones or second-line injectable drugs; or •Adverse drug reactions
Died	A patient who dies for any reason during the course of treatment
Lost to follow-up	A patient whose treatment was interrupted for 2 consecutive months or more
Not evaluated	A patient for whom no treatment outcome is assigned (this includes cases “transferred out” to another treatment unit and whose treatment outcome is unknown)
Treatment success	The sum of Cured and Treatment completed

^a^For Treatment failed, lack of conversion by the end of the intensive phase implies that the patient does not convert within the maximum duration of the intensive phase applied by the programe. If no maximum duration is defined, an 8 months cut-off is proposed. For regimens without a clear distinction between intensive and continuation phases, a cut-off 8 months after the start of treatment is suggested to determine when the criteria for Cured, Treatment completed, and Treatment failed start to apply (for the investigational group, 6 months will be considered as an intensive phase)

Both “cured” and “treatment completed” are defined as treatment success. Treatment failure, death, loss to follow-up, transfer out, and relapse are excluded from treatment success.

### Eligibility criteria

Participants with pulmonary TB satisfying the inclusion criteria are competitively enrolled by investigators in both outpatient and inpatient settings in the 12 participating hospitals. Inclusion criteria are as follows: men and women aged ≥ 19 and ≤ 85 years, with confirmed MDR-TB by phenotypic or genotypic drug susceptibility tests or RR-TB by genotypic tests such as Xpert® *Mycobacterium tuberculosis* (MTB)/resistance to rifampin (RIF) assay (Cepheid, Sunnyvale, CA, USA) regardless of being positive for sputum acid-fast bacilli smear, and use of current anti-TB regimen with second-line drugs for ≤ 14 days at the time of enrollment.

We will exclude patients with any fluoroquinolone-resistant MDR-TB, XDR-TB patients, and pregnant women or women of childbearing age unwilling to use proper contraceptives. Additionally, any of the following factors will lead to exclusion: (1) medical history of galactose intolerance, Lapp lactase deficiency, or glucose-galactose malabsorption; (2) history of optic neuropathy or peripheral neuropathy; and (3) the need for ongoing use of prohibited drugs while on study drugs. Additionally, we will exclude patients having any of the following test results: (1) absolute neutrophil count < 2.0 × 10^3^/μL, (2) white blood cell count < 3.0 × 10^3^/μL, (3) hemoglobin < 7.0 g/dL, (4) serum creatinine > 2.0 mg/dL, (5) aspartate aminotransferase > 100 IU/L, (6) alanine aminotransferase > 100 IU/L, (7) total bilirubin > 2.0 mg/dL, (8) albumin < 2.8 g/dL, and (9) prolonged QT interval (QTc corrected by Fridericia’s formula, QTcF > 500 ms). Finally, patients who have a history of hypersensitivity reaction to the study drugs will be excluded.

### Randomization

We assign participants to the study arm based on a 1:1 randomization ratio. The randomization sequence was generated by a trial statistician using a block randomization method stratified by the presence/absence of cavitation on baseline chest radiographs and the presence/absence of baseline diabetes mellitus. A web-based randomization system is operated remotely at the Medical Research Collaborating Center (MRCC) in Seoul National University Hospital. Access to and management of randomization information will be carried out independently from the tasks of the clinical investigator or trial sponsor.

### Justification of sample size

The hypothesis of this study is that the treatment success rate at 24 months of treatment for MDR-TB using the new regimen including new anti-TB drugs will not be inferior to that of conventional treatment regimens (non-inferiority test).

#### Hypotheses for sample size calculation

The sample size calculation hypotheses are defined as follows.

H_0_ (null hypothesis): *P*_T_ − *P*_C_ ≤ δ (new regimen treatment success rate after 24 months of treatment is inferior to conventional treatment success rate).

H_1_ (alternative hypothesis): *P*_T_ − *P*_C_ > δ (new regimen treatment success rate at 24 months is not inferior to conventional treatment success rate).

#### Assumptions

Based on the results of our previous study, we presume the 24-month treatment success rate of the control arm as 80% [16, 17].

Based on an α = 0.025 level of significance (one-sided test), a power of 80%, < 10% difference in treatment success rates between the control and investigational arms (80% vs. 70%) when we anticipate that the actual success rate in the treatment group will be 90%, the sample size per arm to show non-inferiority of the investigational regimen was calculated to be 48. Additionally, reflecting (1) proportion of fluoroquinolone-susceptible MDR-TB among participants as 50%, and (2) anticipating 5% loss to follow-up, the final number of participants is calculated as *N*/(0.50 × 0.95), resulting in 102 persons/group (204 in total).

If a participant withdraws for any reason within 2 weeks after enrollment, that participant will be replaced. Considering the number of replaced participants, the enrollment accrual ceiling is 220 persons maximum.

### Statistical analysis

The results of this trial for efficacy outcomes will be analyzed based on both modified intention-to-treat (mITT) and per protocol (PP) approaches with a primary consideration for mITT results. A PP analysis will be performed secondarily. A safety analysis will be performed based on the safety group. The mITT group will include participants who are randomized after satisfying eligibility criteria and receive study drugs at least one time. The PP group will include participants who satisfy the following conditions among the mITT group: (1) those who completed > 80% of the planned treatment, (2) those who completed the clinical trial according to the protocol. The safety analysis group will include participants who receive study drugs at least once.

### Efficacy outcomes

Comparisons will be performed using two-sided tests with a statistical significance level of 5% unless stated otherwise.

#### Analysis of primary outcome

For the primary outcome of this trial, we will describe the treatment success rate at 24 months of the control arm and the investigational arm with a two-sided 95% confidence interval. To test for non-inferiority of the investigational arm, when the lower limit of the one-sided 97.5% confidence interval of the difference (*P*_T_ − *P*_C_) between investigational and control arms is larger than the non-inferiority margin of − 10%, it will be concluded that the treatment success rate of the investigational arm shows non-inferiority to the treatment success rate of the control arm.

#### Analysis of secondary outcomes

The analysis of secondary outcomes will be described as exploratory outcomes. To determine whether time to sputum culture conversion after treatment start is statistically different between the control and investigational arms, the median time will be estimated in each group using the Kaplan-Meier method, and the difference in the distribution of time to culture conversion of the two arms will be compared using the log-rank test.

To test whether there is a statistical difference in proportion of sputum culture conversion (liquid and solid culture media) at 2 months or 6 months of treatment, treatment success at the end of treatment, reverting to positive sputum culture after the end of treatment, treatment success according to pyrazinamide resistance, and death between the control and investigational arms, proportions of each arm will be summarized by frequency and percentage and these will be compared using the chi-square test and Fisher’s exact test. The median time to death after treatment start will be estimated in each group using Kaplan-Meier method, and the difference in the distribution of time to death of the two arms will be compared using the log-rank test.

### Safety assessment

All AEs and serious AEs (SAEs) according to the Common Terminology Criteria for Adverse Events (CTCAE), regardless of severity, seriousness, or relationship to the study drug, will be collected and documented.

We will summarize all AEs and SAEs, AE frequency and percentage, and 95% confidence intervals. Additionally, we will summarize and evaluate the occurrence rate of AEs in relationship to the study drug and severity. The occurrence rate of ADEs (CTCAE grades 3 and 4) of the two arms and the proportion per type of toxicity will be compared using the chi-square test or Fisher’s exact test.

### Stratified analysis

Primary and secondary outcomes will be analyzed separately in participants with sputum smear-positive and smear-negative pulmonary TB.

### Data collection and management

This study will use a web-based electronic case report form (e-CRF) with Pharmaco-epidemiology and Clinical Trial Application X (PhactaX). PhactaX has been developed by the MRCC in collaboration with an outsourced contractor. PhactaX is based on Java and Oracle databases and complies with international standards and regulations. The e-CRF designed for this study used dummy variables for user acceptance testing to confirm its validity.

During the study, medical personnel not participating in this study will monitor this trial. Monitors will visit sites to monitor all aspects of the study including adherence to the protocol and Good Clinical Practice, protection of study participants, and data accuracy of the study.

### Supervision of the trial

A Data and Safety Monitoring Board (DSMB) composed of two respiratory specialists who have experience with treating MDR-TB patients and one statistician from another institute with no conflict of interests will be formed. The DSMB will review data every 3 months during the trial and may provide recommendations such as change, continuation, or stopping of protocols to the investigators based on the results.

### Confidentiality

Collection and operation of participants’ personal information will be limited to only information necessary for efficacy, safety, and tolerability evaluation of study drugs. Such data will be collected and processed taking precautions for compliance with laws on privacy protection and guaranteeing of confidentiality. Paper files containing participants’ data (including personally identifiable information and copies of signed consent forms) will be securely stored in a locked office on sites in locked filing cabinets. Digital files containing participants’ data will be stored in password-protected files on university-maintained servers. Access to study files will be restricted to authorized personnel only.

The items in the present study protocol comply with the Standard Protocol Items: Recommendations for Interventional Trials (SPIRIT) checklist (see Additional file 1).

## Discussion

Several trials have been conducted with the aim of overcoming difficulties in MDR-TB treatment. In 2010, a relapse-free cure rate of 87.9% among 206 patients treated with a 9-month regimen of gatifloxacin, clofazimine, ethambutol, and pyrazinamide throughout the treatment period supplemented with prothionamide, kanamycin, and high-dose isoniazid during an intensive phase of 4 months was reported [18]. This result was largely replicated in subsequent studies [19], and WHO endorsed a shorter course regimen in 2016 [5].

However, this shorter regimen includes too many drugs, as many as seven, and still includes an injectable (kanamycin) for the first 4–6 months. Additionally, the number of candidates for this shorter treatment, i.e., patients without resistance to all drugs included in the regimen, could be limited. In European MDR-TB cohorts, on average, only 7.8% were eligible for this shortened regimen [20]. Reports from other areas, including Singapore, Brazil, Pakistan, and South Korea, assume eligibility for the shorter regimen ranged from 30 to 55% [21–24].

Fortunately, repurposed anti-TB drugs including linezolid or newly developed drugs including delamanid and bedaquiline have been introduced for MDR-TB treatment. A meta-analysis of 12 non-randomized studies showed that 82% of patients treated with a linezolid-containing regimen demonstrated favorable treatment outcomes [8]. Additionally, a randomized trial in which linezolid was used for XDR-TB patients reported a 6-month culture conversion rate of 87% and a treatment success rate without relapse at 1 year follow-up of 71% [9, 25]. Delamanid, a nitro-dihydro-imidazooxazole derivative, demonstrated activity against MDR-TB as measured by increased sputum culture conversion rate in a randomized, placebo-controlled trial. Patients who received a background drug regimen plus 100 mg or 200 mg of delamanid twice daily had sputum culture conversion rates in liquid broth at 2 months of 45.4% and 41.9%, respectively, as compared with 29.6% of patients who received a background drug regimen plus placebo [10]. In a subsequent observational extension trial for 24 months, favorable outcomes were observed in 74.5% of patients who received delamanid for ≥ 6 months compared with 55% of patients who received delamanid for ≤ 2 months [26]. Bedaquiline, a diarylquinoline, showed efficacy in a randomized phase II trial; it increased the culture conversion rate to 62% and the cure rate to 58% at 120 weeks as compared with the placebo group (44% and 32%, respectively) [27]. Additionally, bedaquiline-containing regimens achieved high conversion and treatment success rates in a different large retrospective observational study [28].

Based on the proven efficacy of these new anti-TB drugs, several clinical trials using 6–12-month regimens for MDR-TB treatment without an injectable are being tested. First, the STREAM Stage 2 trial (NCT02409290, phase III) is comparing the effectiveness of 6- and 9-month bedaquiline-containing regimens against the conventional WHO regimen and a 9-months regimen including an injectable. Another phase III trial (NeXT, NCT02454205) is testing 6- or 9-month treatments containing bedaquiline, linezolid, levofloxacin, ethionamide/high-dose isoniazid, and pyrazinamide. Additionally, NiX-TB (NCT02333799, phase III) is assessing the safety and efficacy of a 6- or 9-month regimen comprising bedaquiline, PA-824, and linezolid.

The present study, Treatment Shortening of MDR-TB Using Existing and New Drugs (MDR-END), tests a 9–12-month regimen of delamanid, linezolid, levofloxacin, and pyrazinamide for MDR-TB patients without fluoroquinolone resistance. Delamanid and linezolid were selected based on their bactericidal activities in a mouse model [29, 30] and proven effectiveness in MDR-TB patients [10, 17, 26, 31, 32], as well as on limited prior population exposure. In addition, levofloxacin was selected based on bactericidal activity [33], and it demonstrated effectiveness in MDR-TB patients [34] and weaker QT prolongation potential than moxifloxacin [35]. Finally, pyrazinamide was included because of its bactericidal sterilizing activity [15, 36] as well as its confirmed effectiveness in patients with MDR-TB [34].

Showing non-inferiority, although not superiority, of a shorter regimen without an injectable to the conventional regimen for MDR-TB treatment lasting 20–24 months is very important. Poor adherence to treatment is one of the main causes of poor treatment outcome among patients with MDR-TB, and it is strongly influenced by the long treatment duration and use of an injectable, which can cause ototoxicity or nephrotoxicity as well as pain at the injection site. If our trial proves non-inferiority of the 9–12-month fully oral regimen to the conventional 2-year treatment including an injectable, then this shorter regimen could contribute to reducing MDR-TB globally by improving patient adherence to treatment.

## Trial status

Recruitment began at the first site in April 2016 and is expected to be completed by April 2021.

## Additional file

Additional file 1: SPIRIT 2013 checklist: recommended items to address in a clinical trial protocol and related documents. (DOC 127 kb)

## Abbreviations

ADE: Adverse drug event
AE: Adverse event
AFB: Acid-fast bacilli
CTCAE: Common Terminology Criteria for Adverse Events
DSMB: Data and Safety Monitoring Board
DST: Drug susceptibility testing
e-CRF: Electronic case report form
ITT: Intention-to-treat
MTB: *Mycobacterium tuberculosis*
MDR: Multidrug-resistant
MRCC: Medical Research Collaborating Center
PAS: *p*-aminosalicylic acid
PhactaX: Pharmaco-epidemiology and Clinical Trial Application X
PP: Per protocol
RR: Rifampicin-resistant
SAE: Serious adverse event
TB: Tuberculosis
XDR: Extensively drug-resistant
